# Supplementary material for: Are carbon emissions trading and green financial instruments synergistic? -Comprehensive quantitative research based on content analysis
Source: PLoS One. 2024 Mar 7;19(3):e0298601. doi: 10.1371/journal.pone.0298601 (PMC10919601; doi:10.1371/journal.pone.0298601)
Supplement: S1 Data — (ZIP) [file pone.0298601.s001.zip › Data/地级市Z_F工作报告.zip!/╝¬┴╓/│ñ┤║╩╨/└┤╫╘╣1⁄2╓┌║┼í╛┬φ┐╦╩2╛▌═°í┐_2013.docx]

各位代表：

　　现在，我代表市人民政府，向大会报告工作，请各位代表审议，并请市政协委员提出意见。

　　一、2012年和本届政府工作回顾

　　2012年是极不寻常的一年。在市委的坚强领导下，在人大、政协的监督支持下，我们紧紧依靠全市人民，坚定信心，迎难而上，顽强拼搏，从容应对复杂多变的外部环境，成功保持了经济社会持续健康发展的良好态势。

　　预计地区生产总值完成4506.6亿元，增长12%；工业增加值完成1997.5亿元，增长12.5%；服务业增加值完成1854.5亿元，增长12.1%；固定资产投资完成3100亿元，增长27.4%。这些主要经济指标的增长速度在15个副省级城市中均排在前列。

　　实现全口径财政收入927亿元，增长15.5%；地方财政收入333亿元，增长15.5%；规模以上工业企业利润630亿元，增长23%；城市居民人均可支配收入23089元，增长12.7%；农民人均纯收入8570元，增长12.1%。这些体现经济增长质量和群众收入水平的指标均与经济实现了同步增长。

　　实践表明，长春经受住了困难和风险的考验，在全面振兴的道路上又迈出了新的坚实步伐。

　　一年来，我们主要做了以下工作：

　　（一）积极克服下行压力，经济增长实现稳中有进

　　先后出台25项稳增长政策，连续采取促进自主品牌汽车销售、壮大战略性新兴产业等一揽子措施，不仅稳定了经济大局，还促进了结构调整。

　　全年生产汽车185万辆，增长13.4%，完成产值4910亿元，增长17.5%，这两项指标增速分别高于全国8.8个和4.8个百分点。

　　农产品加工业产值增长29.5%，为工业增长做出了突出贡献。

　　先进装备制造、光电信息、生物医药、新能源、新材料五大战略性新兴产业产值突破千亿元大关，科技创新引领发展作用日益显现。

　　现代物流产业增加值增长18.5%，金融服务业增加值增长21%，文化产业增加值增长40%，旅游业总收入增长27%，现代服务业占经济比重稳步提高。

　　引进富士康等9户世界500强、国内500强企业，实际利用内外资均增长20%以上。

　　农业战胜春旱、虫灾、台风灾害喜获丰收，粮食产量达到220亿斤，再创历史新高。

　　在全省县域综合发展考评中，四县(市)全部进入综合排序前十名，农安县连续两年位列第一。

　　（二）全力加快项目建设，城市发展后劲明显增强

　　“十二五”规划确定的5大类150个重大项目按预定计划顺利推进，全市投资3000万元以上项目总数达到2480个，比上年增加400余个。

　　城市中心区，实施了300多个投资亿元以上项目，推动服务业全面扩容、提档升级。

　　城市西南，一汽丰越10万辆整车竣工投产，轿股三厂50万辆整车、轴齿工业园、乘用车研发中心等重大项目相继开工，世界级汽车产业集群正在崛起。

　　城市西北，36个轨道客车配套项目开工建设，建筑面积28万平方米的轨道客车装备园区初具规模。

　　城市东北，北远达大街、北湖大桥建成通车，打通了连接城市中心区的两条主干线，引进并建设了67个战略性新兴产业项目，总投资额同比提高近一倍。兴隆保税区具备封关运营条件，首批落位18个外向型项目，长春对外开放的新高地正在形成。

　　城市东部，莲花山度假区、二道物流产业园区、九台空港新城、长德新区竞相建设、齐头并进，长吉一体化步伐不断加快。

　　净月西部，直通新立城坝下的生态大街全线贯通，重大项目纷纷落位，总长15公里的高端服务业产业带已现雏形，正在全力建设创新型生态城。

　　（三）努力加大投入力度，城市建设改造全面铺开

　　今年，城市建设实施了78项重点工程，资金投入达到408亿元，相当于上年5.4倍。

　　“两横三纵”快速路全线开工，亚泰大街跨繁荣路、自由大路高架桥投入使用，机场大道建设顺利推进，三环路开始拓宽改造，初步构建起立体化的城市道路主框架。

　　轻轨4号线投入运营，地铁1号线转入全程施工，地铁2号线开工建设，长春西站综合换乘中心、长春站综合换乘中心北枢纽投入使用，长春大踏步地迈向轨道交通时代。

　　长平高速改扩建以及长春至双辽、九台至双阳高速公路开工建设，哈大高铁正式投入运营。

　　新建扩建4座净水厂、5座污水处理厂。伊通河城区段截污干管完成改造，串湖水系及3条城市明沟实现污水截流。改造燃气危旧管网340公里、供热管网311公里、供水管网264公里、排水管网130公里。新增供热能力2799万平方米。主次街路机械化清扫率由21.4%提升到47.2%。

　　新建续建7座公园，9.7平方公里的长东北湿地公园正式对游人开放。改造精品绿地63处，绿化彩化街路121条，新建城市绿地500余公顷。

　　新一轮土地利用总体规划通过国务院审批，新增建设用地指标4.82万公顷。

　　强化交通管理，在全年新增12万辆新车、城市道路改造全面铺开的情况下，城市交通基本保持通畅。

　　深入开展市容环境综合整治，城市管理水平不断提高。

　　（四）坚持民生第一目标，切实办好惠民利民实事

　　今年，我们把市级新增财力70%以上用于改善民生，为群众办了100余件实事。

　　创业促就业工作取得突出成效，被国务院命名为全国创业先进城市。

　　提高了职工最低工资标准、个体工商户个税起征点、退休人员养老金标准以及“三无”人员、农村五保户供养标准。

　　新增农村居民、城市居民、城镇职工养老保险参保人数170余万，养老保险扩面取得历史性突破。

　　为4.9万户低保群众发放“幸福惠民卡”，低保救助力度进一步加大。

　　建立了贫困家庭子女从学前教育到高等教育全程资助的政策体系，力争不让一个孩子因贫失学。

　　积极开展听障、脑瘫、孤独症儿童康复医疗救助，一批残疾人家庭实施无障碍改造，残疾人康复中心启动建设。

　　新建保障性住房10645套。2123户低保家庭入住廉租房。200万平方米无籍房完成确权。1221万平方米老旧楼宇完成“暖房子”改造。600余栋住宅完成楼道粉刷。拆除D级危房117栋。综合整治老旧散住宅区33个。住房公积金覆盖面稳步提高。

　　深入创建幸福社区，社区管理和公共服务能力进一步增强。

　　提高了职工医保、居民医保和新农合报销封顶线，新增20个定额治疗病种，70余项常规诊疗项目费用平均降低23%。全面推广五城区成功经验，四县（市）和双阳区中心医院启动基本药物制度改革。率先在全国免费开展乙肝病毒母婴阻断工程。市中医院开工建设，市中心医院扩建顺利推进，市心理医院建成投入使用。首轮健康长春行动计划全面完成。国家卫生城市复检顺利通过验收。

　　新建改建38所公办幼儿园，增加普惠性幼教学位5500个。孤独症儿童康复教育中心、2所民族中小学、4所城乡接合部学校建成投入使用，40所中小学完成校舍、操场、消防设施标准化改造。开展“大学区”改革，推动义务教育学校资源共享、优势互补，稳步提高教育均衡水平。大力发展职业教育，合隆职教园区前期准备顺利推进，农业学校异地新建项目一期工程竣工。新购657台标准校车，4.5万名农村中小学生获得安全校车服务。

　　深入开展国家公共文化服务体系示范区创建，孔子文化园建成开放，市档案馆完成改造，市图书馆改造顺利推进，新建改建7个县级图书馆、文化馆，114个乡镇（街道）综合文化站、1320个村（社区）文化活动室达到国家规定标准，308个社区、841个行政村配备文化专干，公共图书馆、文化馆、乡镇（街道）综合文化站实现免费开放。市县两级文化体制改革顺利完成。

　　圆满完成第十二届全国冬季运动会长春赛区承办任务，以29块金牌的成绩荣列金牌榜第二名。奥林匹克公园3个体育场馆完成主体工程。

　　（五）加强和创新社会管理，社会大局保持和谐稳定

　　自觉接受人大及其常委会的法律监督、工作监督和政协的民主监督，坚持重大事项向人大报告、与政协协商制度。认真听取各民主党派、工商联以及无党派人士的意见建议。办理人大议案4件、人大代表建议191件、政协提案287件。

　　按照“人性化、网格化、信息化”的要求，夯实基层基础，创新社会管理服务体系，积极化解社会矛盾，强化为民服务能力。

　　深入开展“安全建设年”活动，排查整改安全隐患2.2万处，安全生产事故发生起数、死亡人数、经济损失分别下降43.5%、20.4%和49.6%。

　　深入开展平安创建活动，严厉打击各类刑事犯罪，110有效警情、刑事案件立案数分别下降31%和13.3%，命案破案率达到95.9%，群众安全感进一步提升。

　　深入开展食品安全专项整治和“百日会战”，食品安全监管取得新成效。

　　充分利用信访机构接待、市长公开电话、局长接待日、读报读网制度等各种渠道，及时掌握群众诉求，主动解决群众困难，群众信访总量降低12%。

　　扎实开展“三满意”机关建设和“万人评议机关”活动，经济发展软环境进一步改善。

　　强化依法治市，严格依法行政，坚持从严治政，政府自身建设取得新进展。

　　深入开展双拥共建，实现全国双拥模范城“七连冠”。

　　国家安全、工商、质监、统计、民族、宗教、外事、侨务、保密、人防、气象、地方志、红十字等工作都取得了新成绩。

　　各位代表，今年我们取得的丰硕成果，标志着长春经济社会发展跃上了一个新台阶，为本届政府五年任期画上了圆满句号。

　　自2007年市十三届人大一次会议以来的五年，长春不仅肩负着老工业基地调整改造的艰巨任务，而且遭遇了国际金融危机影响的严重冲击。全市人民战胜重重困难大踏步前行，经济社会发生了前所未有的深刻变化。

　　过去的五年，是综合经济实力全面跃升的五年。地区生产总值比2007年增长1.2倍，全口径财政收入增长2.2倍，规模以上工业总产值增长1.9倍，固定资产投资增长1.3倍，进出口总额增长1.5倍。在15个副省级城市中，经济增长速度始终位居前列，全口径财政收入由第14位前移到第12位，规模以上工业总产值由第12位前移到第9位，固定资产投资由第13位前移到第11位。主要指标翻番式增长、位次明显前移，标志着长春踏上了全面振兴的新征程，进入了大发展、快发展的新阶段。

　　过去的五年，是改革开放攻坚突破的五年。国有企业完成改制攻坚任务，国企改革遗留问题基本得到解决。民营企业由3.5万户增加到5.4万户，新增纳税超千万元民营企业230余户。先后与5个外国城市建立友城关系。引进13家世界500强、227户外资企业，外资企业税收占工业税收比例达到1/3以上。高新区、经开区新区建设取得实质性突破，汽车区、净月区晋升为国家级开发区，19个省级开发区迅速发展壮大，开发区进入了新一轮快速成长期。改革开放为长春发展注入了强大活力，也为未来奠定了坚实基础、拓展了广阔空间。

　　过去的五年，是产业结构不断优化的五年。200万辆整车、2000辆轨道客车、百万吨化工醇扩能工程顺利完成，三大世界级产业基地初具规模。国家创新型城市建设取得明显进展，五大战略性新兴产业迅速发展壮大，成为支撑长春经济增长的新生力量。服务业投资连续5年位居三次产业之首，对经济增长的贡献率持续递增。长春已经基本形成了多业支撑、多业并举的产业格局，经济运行的稳定性和抗波动能力显著增强。

　　过去的五年，是农业农村蓄势变革的五年。新增粮食综合生产能力30亿斤，3个县（市）稳居全国十大产粮县行列，榆树连续9年产粮全国第一。农机化综合水平稳步提高，解放了农村劳动力，促进了土地规模经营。农产品加工业成为支柱产业，工业在县域经济中开始发挥主导作用，县（市）财政收入增长近4倍。农村人口开始向城镇集聚，县城人口均达到中等城市规模。现在的县域，以农耕为主的传统模式正在快速转型，初步形成了工业化、城镇化、农业现代化加速推进、良性互动的新局面。

　　过去的五年，是城市面貌日新月异的五年。先后启动了南部新城、西部新城、长东北、莲花山等区域开发建设，进一步加快北部新城、西南工业区改造开发，建成区每年以15平方公里以上的速度快速扩张。基础设施建设扎实推进，道路面积增长1.2倍、发电能力增长1.3倍、供热能力增长1.5倍、天然气供应能力增长1.4倍。29个公园、460余处绿地完成新建改造，30余条主要街路实施综合整治，2000余万平方米棚户区变成了环境优美、功能齐全的现代化居住区。2008年长春被命名为国家卫生城市，2011年进入全国文明城市先进行列，城市综合质量显著提升，正向绿色宜居城市的目标阔步前进。

　　过去的五年，是社会事业繁荣进步的五年。医疗卫生体制改革取得突破性进展，率先在全国实行医疗保险付费方式改革，公办基层医疗机构全部完成标准化改造并实施了基本药物制度。基本完成中小学校舍安全改造，学前教育、基础教育、职业教育、特殊教育长足发展。修缮改造沙俄领事馆、南大营抗战陈列馆、南广场等历史建筑和历史街区，新建改造科技文化中心、光学博物馆、艺术剧场、杂技宫等文化设施。广场和社区演出、数字电影放映、送戏下乡等活动已成常态，群众文化生活日益丰富。全民健身活动蓬勃开展，冬夏奥运会均实现金牌零的突破。

　　过去的五年，是民生状况显著改善的五年。坚持每年召开一次民生大会、出台一个民生行动计划、为群众办100件左右实事。五年城镇新增就业45万人，城镇登记失业率始终控制在4%以内。职工最低工资、社会平均工资、离退休人员养老金都提高了80%以上。养老、失业、工伤、生育保险和住房公积金参保人数均增加70%以上，城乡医疗保障基本实现全覆盖。4次提高城乡低保标准、困难劳模补助标准，及时根据物价上涨情况向困难群众发放生活补贴。贫困家庭精神病人、白内障患者、唇腭裂儿童康复医疗救助基本实现“有一助一”。二次供水设施不达标、居民小区临时用电、退休职工进医保、困难群体接续养老保险等历史遗留问题的解决使60余万群众受益。棚户区改造、保障房建设、泥草房改造、“暖房子”工程帮助100余万中低收入群众改善了居住条件。民生工作得到了群众的认可和社会的高度评价，长春连续5年被评为最具幸福感城市，全市人民在更加和谐的社会环境中不断增进福祉、共享发展成果。

　　各位代表，在过去五年发展实践中我们深刻地体会到：只要我们在市委的坚强领导下，坚定方向、坚持不懈，就一定能够战胜各种困难和险阻；只要我们自觉接受人大、政协的监督，汲取力量、争取支持，就一定能够科学民主决策；只要我们把人民群众的利益高高举起，以人为本、执政为民，就一定能够赢得可靠持久的动力源泉。在此，我代表市政府，向为长春发展做出突出贡献的全市人民致以崇高的敬意！向给予我们监督与支持的人大代表、政协委员和社会各界人士，向积极参与长春发展建设的中省直驻长单位，人民解放军和武警驻长部队以及海内外的朋友们，表示衷心的感谢！

　　成绩鼓舞人心，经验弥足珍贵。我们也清醒地认识到，经济社会发展和政府工作还面临着许多困难和问题。主要是：经济总量仍然偏小，产业结构不尽合理，自主创新能力不强，资源环境矛盾突出，部分群众生活仍然比较困难，社会还存在一些不稳定因素，政府管理和服务水平有待提高，廉政建设需要进一步加强。这些问题都需要我们在今后工作中高度重视并着力加以解决。

　　二、未来五年主要任务

　　党的十八大、省十次党代会、市十二次党代会，为我们明确了未来五年的主要任务，这就是建设幸福长春，率先全面建成小康社会，让城乡居民生活得更加美好。

　　为完成这样的任务，我们要坚持以邓小平理论、“三个代表”重要思想、科学发展观为指导，紧紧围绕主题主线，围绕科学发展、改善民生、建好城市、促进和谐大局，进一步实施好“三化”、“三动”、“三大板块”协调发展、工业和服务业“双拉动”等重大战略，以提高经济增长质量和效益为中心，坚持稳中求进总基调，促进经济持续健康发展，加快调整产业结构，积极稳妥推进城镇化，深化改革开放和创新驱动，着力保障和改善民生，促进社会和谐稳定，切实把经济建设、政治建设、文化建设、社会建设、生态文明建设的各项任务落到实处。

　　实际工作中，我们要采取以下措施：

　　——推动经济转型增长。我们不仅要连续保持不低于副省级城市平均水平、全省平均水平的增长速度，还要更加重视质量、更加重视效益、更加注重科技创新，在不断转变发展方式、优化经济结构、提高发展可持续性的基础上，力争实现地区生产总值和地方财政收入翻番，人均GDP达到15000美元以上，综合经济实力进入中等发达城市行列。

　　——大力调整经济结构。我们要调整城乡结构，稳步提高城镇化率和城镇化质量，初步形成城乡一体化发展格局。调整产业结构，充分发挥科技创新对产业升级的支撑引领作用，做精一产、做强二产、做大三产。调整工业结构，加快建设汽车、轨道客车、农产品加工三大世界级产业基地，推动战略性新兴产业在全国形成较强的竞争力。调整投资与消费结构，在发挥投资对经济增长关键作用的同时，逐年提高消费对经济增长的贡献率。

　　——加强城市建设管理。我们要保持城市化的旺盛势头，高标准建设“三城两区”，构建“双心、两翼、多组团”的城市空间结构。建设快速路、轨道交通、现代公交和慢行交通系统，形成能够承载百万辆汽车的现代交通体系。全面改造地下管网设施，确保水、电、气、热等生产要素能够满足当期需求并适度超前。基本完成夹馅棚户区、危旧房屋、历史文化街区改造，切实加强城市管理，实现城市形象全面提升。

　　——统筹“三大板块”发展。我们要坚定不移地支持开发区转型升级、率先增长、带动发展，加速成为以新型工业化、高端服务业为主要产业内容的现代化新城区。各城区要成为服务业的主战场，在环境建设、城市管理、民生改善、社会和谐等各领域实现全面进步。各县（市）都要成为中等城市，尽快形成以二、三产为主的经济结构，人口加速向城镇集聚，社会主义新农村建设全面推进。

　　——强力推进改革开放。我们要以更大的勇气和智慧，不失时机深化重要领域改革，坚决破除一切不合时宜的束缚和桎梏。不断深化国有企业体制机制创新，推动国有资本更多地投向公益性行业和关键领域。支持和引导民营经济加快发展，完善中小企业发展政策，善待和支持小微企业。实施更加积极主动的开放战略，吸引国内外资金、技术、人才向长春快速集聚，全面提升开放型经济水平。

　　——全力改善生态环境。我们要加快建设城市森林、城市湿地、城市水域、城市绿地，不断加大自然生态系统修复保护力度。全面强化节能减排，能源、土地、水消耗强度分别降低30%以上，主要污染物排放总量下降50%以上。增强全民节约意识、环保意识、生态意识，积极推行循环经济、低碳经济等绿色经济模式，努力把长春建设成为天蓝、地绿、水净、城美的绿色宜居森林城。

　　——推进社会管理创新。我们要加强基层社会管理和服务体系建设，引导社会组织健康有序发展，充分发挥群众参与社会管理的基础作用。畅通和规范群众诉求表达渠道，建立健全重大决策社会稳定风险评估机制，从源头减少和化解社会矛盾。健全民主法制，保障公共安全，提高社会公平正义水平，努力开创社会和谐人人有责、和谐社会人人共享的生动局面。

　　——提高人民生活水平。我们要实施更加积极的就业政策，努力让更多群众实现更加稳定、更高质量的就业。大力推动富民增收，力争城乡居民收入实现倍增，群众收入差距不断缩小，中等收入群体持续扩大。加快完善基本公共服务体系，努力办好人民满意的教育，群众健康水平明显提高，基本文化权益得到更好保障，保障性住房力争应保尽保，城乡养老保险基本实现全覆盖，人人享有基本生活保障。

　　各位代表，长春人民为了生活得更美好，始终在不懈地努力、顽强地奋斗。现在我们比以往任何时候都更接近这个目标。让我们携手并肩、戮力同心，坚定地朝着这个目标奋勇前进。长春人民应该过上更美好的新生活，也一定能够过上更美好的新生活。

　　三、2013年重点工作

　　明年是新一届政府的开局之年。长春发展外部环境有向好的趋势，但我们要对困难有清醒的认识，做充分的准备，争取最好的结果，确保新一届政府各项工作顺利起步、扎实开局。

　　全市经济社会发展主要预期目标是：地区生产总值增长13%左右，地方财政收入、城乡居民收入与经济保持同步增长，固定资产投资增长25%以上，工业增加值增长14%，进出口总额增长15%，社会消费品零售总额增长15%左右。

　　实际工作中，我们不仅要保持经济持续健康发展的好势头，取得质量效益同步提升的好成绩，还要在转变发展方式上取得新进展，在改革开放上取得新突破，在改善民生上取得新成效。

　　（一）突出增量调整，做大做强工业

　　明年，我们要坚持大上工业不动摇，依靠增量推动工业转方式、调结构、提质量。

　　狠抓工业项目建设，一汽轿股三厂50万辆整车、轴齿工业园、大成老厂区搬迁、长客装备等重大项目取得实质性进展。积极吸引世界500强、国内500强等战略投资者来长投资，在建投资10亿元以上工业项目数量达到50个，3000万元以上工业项目达到1000个，工业投资增长20%以上。

　　大力发展产业集群，汽车区核心零部件、经开北区生物化工、宽城和绿园轨道客车、净月汽车电子、高新北区战略性新兴产业、皓月清真产业要尽快达到千亿元产值规模。

　　加快建设创新型城市，强化“四位一体”创新驱动联盟，新建科技孵化器50万平方米，转化重大科技成果100项以上，战略性新兴产业增加值增长20%以上。突出企业科技创新主体作用，尽快壮大光显示、激光技术、半导体照明、稀土镁合金等重点产业，使之成为长春未来发展的重要支撑。

　　实施骨干企业成长工程，努力培育一批产值超百亿元、超500亿元的大企业。全面加大对中小企业的扶持力度，年内新增规模以上企业50户、中小企业1500户。

　　加快调整产品结构，提高产品附加值，抢占产业链中高端，工业增加值率提高2个百分点以上。大力实施质量强市、商标品牌战略。

　　（二）突出融合互动，强化动力支撑

　　明年，我们要更加重视服务业，积极促进工业、服务业深度融合、良性互动，加快构建“双拉动”的发展格局。

　　加快建设长东北核心区、南部新城核心区、净月生态大街三大高端服务业聚集区，在建项目力争达到80个以上。北部新城要加快构建环铁商圈。经开南区部分区域要“退二进三”。长春西站周边、汽车区汽车公园周边要尽快启动服务业开发。莲花山度假区高端生态旅游开发要出形象、出进度。各城区要启动建设一批商业中心、特色商业街区，完善商贸流通体系，推动传统服务业晋档升级。

　　支持国内外企业在长设立区域性总部、研发中心、数据中心。新建续建4家五星级酒店、8家区域性批发市场。积极引进域外金融机构。大力支持企业上市融资。

　　加快建设区域性教育中心、医疗中心、购物中心、旅游中心、会展中心，集聚省内外高端消费。加快培育一批拉动力强的消费增长点，消费对经济增长贡献率提高2个百分点。

　　全面推进新一代信息技术应用，建设数字城市、无线城市、智慧旅游城市。大力发展软件与服务外包、电子商务、云计算数据平台等现代高端服务业。

　　启动建设长东北铁路物流园区，加快完善汽车物流通道，不断提升物流服务功能。

　　（三）突出城乡统筹，加快县域突破

　　明年，我们要以工促农、以城带乡，推动城乡一体化发展，努力开创县域发展振兴的新局面。

　　推动农安合隆、德惠米沙子、九台卡伦等县域开发区尽快与主城区融为一体，承接产业、吸纳就业、集聚人口、建设新城，成为拉动县域发展的强大引擎。长德新区要完成3平方公里项目摆放，经九合作区要启动建设，尽快形成国家级开发区带动县域发展的新格局。

　　四县（市）要以更大的力度推进工业化、城镇化，都要培育有特色的工业集群，都要按照中等城市标准抓好县城建设，都要突出培育一批重点乡镇。

　　启动新一轮粮食增产工程，逐步推广高光效农业，建设50万亩标准化粮田，玉米保护性耕作面积达到150万亩。推广农业机械深松。进一步提高农机化综合水平。创新农业经营体制。积极引导城郊退出普通粮食种植，向都市农业转型。抓好“菜篮子”工程，当年新增棚膜菜田2万亩。实施现代畜牧业提升计划，建成标准化养殖示范区200个。大力发展农业信息化。严格保护耕地，强化节约集约用地。

　　建立农业产业化发展基金，扶持农产品加工企业，农产品加工业增加值增长10%以上。

　　大兴农田水利，改造3处大型灌区、22处中型灌区，推进德惠五大围堤加固工程，完成10座病险水库除险加固。

　　启动幸福乡村建设计划，新建160个省级新农村示范村，绿化美化村屯200个，更新改造农防林600公顷，新建科学储粮仓5万套，解决20万农村群众饮水安全问题。

　　（四）突出建管并重，提升城市形象

　　明年，我们要以更大的力度推动城市建设、强化城市管理，力争城市绿色宜居水平有新的明显提高。

　　“两横三纵”快速路力争主线通车，机场大道城区段投入运行，三环路拓宽改造以及吉林大路、南湖大路东延长线等骨干工程取得实质性进展。加快建设腾飞大路跨绕城高速等立交桥。打通50条卡脖路、断头路。

　　续建地铁1、2号线，启动建设轻轨3号线东延长线、北湖快轨，54路有轨电车延伸线力争投入运营。

　　加快扩建一、三水厂，完成二、四水厂升级改造，启动建设六水厂。新建第五热电厂，供热能力新增1000万平方米。改造供热管网200公里、燃气危旧管网240公里。加快升级城市电网。新建生活垃圾焚烧发电厂、餐余垃圾处理厂。

　　新建续建4座污水处理厂，力争3座污水处理厂建成投入使用。实施伊通河城区段雨污分流改造，启动新凯河、西部串湖和城市明沟生态综合整治，尽快形成美丽、生态、清澈的城市水系。

　　新建续建17座公园，新植街路40条、绿化彩化街路100条，新增城市绿地600公顷。

　　深入开展市容环境综合整治，从严治理城市管理顽疾，提高精细化管理水平。

　　伴随城市建设改造高峰的来临，明年城市交通拥堵压力将有增无减。我们要采取一切可以采取的措施，保畅通、保运转、保出行，全力克服暂时的困难，确保平稳有序地度过这段阵痛期。

　　（五）突出健全体系，推动文化繁荣

　　明年，我们要以健全基本公共服务体系为突破口，加快提升文化软实力，更加主动地迎接城市文化的大发展、大繁荣。

　　推动文化和科技融合，大力扶持文化创意、数字出版、影视传媒、动漫游戏等新兴行业和新型业态，壮大知合动漫、净月广告创意等一批文化产业园区，扶持100户文化骨干企业，尽快把文化产业培育成新的支柱产业。

　　加快推进市博物馆建设，新建群众文化活动中心、朝鲜族群众艺术馆、市图书馆铁南分馆，完成长影老厂区、方志馆改造，长影世纪城二期对外开放，县、乡、村文化基础设施全部达到国家规定标准，确保通过国家公共文化服务体系示范区验收。开展全民阅读活动，举办广场文化活动200场，放映公益数字电影22000场以上。

　　不断深化文化体制改革，加快构建有利于文化繁荣发展的体制机制。

　　广泛开展全民健身活动。社区健身路径实现全覆盖，为10个乡镇、100个行政村安装健身器材。体育职业技术学院新建工程竣工，奥林匹克公园部分投入使用。

　　（六）突出改善民生，强化公共服务

　　明年，我们要围绕增收富民、保障惠民、实事利民三大工程，制定实施幸福长春行动计划，努力为长春群众创造更多的福祉。

　　增收方面，新增城镇就业12万人，农村劳务输出100万人次，应届高校毕业生就业率达到85%以上，“零就业家庭”保持动态为零，城镇登记失业率控制在4%以内。健全职工工资正常增长、集体协商和支付保障机制，努力增加职工收入。强化创业培训，增加创业贷款，力争新增小微型企业5000户、个体工商户2万户，推动创业成为富民的重要途径。多渠道增加居民财产性收入。

　　养老方面，积极推动城镇职工、城乡居民进入养老保险体系，力争年内实现城乡养老保险基本全覆盖。进一步提高养老金发放标准。加快建设第二福利院，回族福利院投入使用。增加非营利性养老机构建设补贴和运营补贴，鼓励社会力量兴办养老服务机构。采取政府购买服务的方式为生活困难的居家老人提供日常养老服务。

　　救助方面，深入开展“万户特困户结对救助”活动。进一步提高城区最低生活保障标准、失业保险金发放标准和“三无”人员、农村五保户供养标准。向城乡低保和低保边缘家庭中小学生发放营养午餐补贴。城区17岁以下贫困听障少年助听器配发、贫困残疾人家庭无障碍改造基本实现“有一助一”。制定出台城镇贫困残疾人参加养老保险优惠政策。残疾人康复中心完成主体工程。

　　教育方面，全面实施基础教育质量提升工程。新建改建15所公办幼儿园，新增普惠性幼教学位4000个。积极扶持民办幼儿园特别是普惠性民办幼儿园发展。规范幼儿园办学行为。提高义务教育生均公用经费，推动办学设施标准化，加强教师队伍建设，创建新优质学校。实施新一轮特殊教育三年规划。积极推动国家高职改革试点，合隆职教园区主体工程竣工，农业学校新校区投入使用。

　　健康方面，启动实施第二轮健康长春行动计划，创建国家健康促进示范城市。加快推进儿童重症诊疗中心、市中医院、市中心医院建设改造工程。在部分市级公办医院推广基本药物制度。大幅度提高职工医保、居民医保门诊报销限额。建立儿童白血病、血友病、再生障碍性贫血等10个重大疾病定额治疗制度，超出定额费用由医保全额报销。建立城镇居民大病保险制度，完善新农合大病二次补助制度，设立贫困群众大病救助专项资金，减轻群众大病医疗支出负担。加强全科医生培养, 提高基层医疗卫生服务水平。

　　居住方面，加快建设保障性住房，年内力争竣工1万套。完成老旧楼宇“暖房子”改造1000万平方米。加大D级危房改造力度。确权无籍房200万平方米。综合整治老旧散住宅区30个。下大气力解决居民小区弃管问题，力争用三年左右时间实现基本物业管理全覆盖。充分发挥住房公积金作用，帮助中低收入家庭改善住房条件。

　　出行方面，新建农村公路1000公里以上，确保村村通、屯屯通覆盖率分别达到100%和60%。建立村村通公路维修养护长效机制，力争三年内道路完好率提高到90%以上。改造农村公路危桥险桥，力争两年内全部达到安全标准。改造城区巷道1000条，四环内街路路灯基本实现全覆盖。新建公交候车亭100个，新购公交车500台以上，进一步增加公交专用道，建设智能公交管理系统，全面提升公交服务水平。

　　（七）突出改革创新，加强社会管理

　　明年，我们要以改革创新为统领，全面强化社会管理体制机制建设，努力营造既充满活力又和谐稳定的社会环境。

　　按照“人性化、网格化、信息化”的要求，不断加强和创新社会管理、公共服务体系。进一步简政放权，强化区级政府社会管理职能。积极推动街道办事处转型，把主要精力转到社会管理和改善民生上来。全面创建幸福社区，努力提高社区服务能力，城区社区公共服务用房面积三年内力争全部达到1000平方米。培育社会组织，强化居民自治。

　　继续开展“大排查、大走访、大接访”活动，不断完善联合接访机制，充分利用市长公开电话等各种载体，畅通诉求表达渠道，排查化解民生难题。推行政务公开，加强行风建设。

　　深入开展“安全建设年”活动，全面排查整治生产、交通、消防等领域安全隐患，坚决遏制重特大事故发生。加强防灾减灾基础设施建设，逐步完善应急物资储备和联动救援机制。

　　建立食品安全质量可追溯体系，批发市场、农贸市场、大型超市食品快速检测基本实现全覆盖，强化食品安全监管，创建食品安全城市。

　　深入开展平安创建活动，落实社会治安打、防、控措施，严厉打击各类刑事犯罪活动，坚决做到黄赌毒必治、黑恶势力必除，命案破案率保持在90%以上，不断提高人民群众安全感。

　　推进“六五”普法和法律服务惠民行动。进一步做好国家安全和民族宗教工作。

　　创新开展双拥共建，推动军民融合式发展，为争创全国双拥模范城“八连冠”打下坚实基础。

　　各位代表，为了肩负起建设幸福长春这一艰巨而光荣的历史重任，我们必须高度重视并切实加强政府自身建设。

　　一定自觉接受人大及其常委会的法律监督、工作监督和政协的民主监督，坚持依法行政，努力建设法治型政府。

　　一定认真学习党的方针政策、各种专业知识，适应不断变化的新形势，努力建设学习型政府。

　　一定奋发有为地努力工作，狠抓工作作风转变，狠抓各项工作落实，努力建设务实型政府。

　　一定全心全意为群众谋福祉，始终与长春人民同甘共苦、团结奋斗，努力建设服务型政府。

　　一定加大反腐倡廉力度，做到干部清正、政府清廉、政治清明，努力建设廉洁型政府。

　　各位代表，幸福长春的美好前景鼓舞人心，全面建成小康社会的宏伟蓝图催人奋进。在新的五年征程里，让我们紧密团结在以习近平同志为总书记的党中央周围，在市委的坚强领导下，满怀激情，锐意进取，为建设幸福长春、让全市人民生活得更加美好而努力奋斗！
